# Cannabis-Impaired driving: ethical considerations for the primary care practitioner

**DOI:** 10.1080/07853890.2022.2151716

**Published:** 2022-11-29

**Authors:** Katherine Huerne, Carolyn Ells, Roland Grad, Kristian B. Filion, Mark J. Eisenberg

**Affiliations:** a Lady Davis Institute for Medical Research, Jewish General Hospital, Montreal, Canada; b Department of Medicine, Division of Experimental Medicine, and Biomedical Ethics Unit, McGill University, Montreal, Canada; c Department of Family Medicine, McGill University, Montreal, Canada; d Departments of Medicine and of Epidemiology, Biostatistics and Occupational Health, McGill University, Montreal, Canada; e Division of Cardiology, Jewish General Hospital, McGill University, Montreal, Canada

**Keywords:** Critical interpretive review, ethical analysis, cannabis-impaired driving, primary care practitioners

## Abstract

**Background:** Widespread cannabis consumption and recreational cannabis legalization is thought to have led to an increase in motor vehicle accidents, although there currently lacks ethical guidance for primary care practitioners on cannabis-impaired driving.

**Objective:** The aim was to develop an ethical framework for primary care providers on cannabis-impaired driving.

**Methods:** An ethical analysis in the form of a critical interpretive review was undertaken, using a systematic approach to determine the appropriate action to a given situation with evidence to substantiate its claims. The search strategy was designed to answer the research question: What are some ethical concerns for primary care providers to consider when cannabis-impaired driving is suspected? Four databases were searched in December 2021 using keywords related to cannabis, impaired driving, ethics, and primary care. The resulting evidence was synthesized as recommendations for primary care practice.

**Results:** The ethical approach for primary care practitioners in addressing cannabis-impaired driving can be summarized as the duty to always inform, provide care through prevention and harm reduction strategies, and report when necessary. The prevention of cannabis-impaired driving should not fall on the sole responsibility of primary care practitioners. As this review offers a high-level discussion of the ethical considerations in cannabis-impaired driving, specific recommendations will depend upon the legal and policy designations of individual jurisdictions.

**Conclusion:** Ultimately, the practitioner should manage cannabis-impaired driving in a way that fosters the therapeutic relationship in patient-centered care, through motivational discussions, collaboration with specialists, skills for self-management, patient empowerment, and support.

**KEY MESSAGES**
**  Take-Home Points for Primary Care Practitioners in Cannabis-Impaired Driving**
  •  For patients who report driving frequently and using cannabis, the frequency of use, dosage, form of cannabis, tolerance levels, and withdrawal symptoms should be discussed, while informing the patient of the risks, harms, and legal consequences associated with cannabis-impaired driving.
  •  The practitioner’s primary responsibility in the cannabis-impaired driving context is to provide care to patients who drive and consume cannabis, which may include referring patients to mental health care to manage addictive or problematic behaviors associated with cannabis use.
  •  Practitioners may have a duty to report cannabis-impaired driving to legal authorities (such as law enforcement) when the user engages in harmful behavior to themselves or others.

## Introduction

With recreational cannabis use increasing in many jurisdictions as a result of its legalization, the intersection of drug use and road safety has become an increasing public health concern [1,2]. There are well-documented sensory, motor, and cognitive impairments that arise from the psychoactive effects of cannabis, such as delayed reaction time in automobile operation [3–5]. Although the magnitude of detrimental effects is debated, it is nonetheless important to prevent such consequences [6]. Primary care providers (e.g. physicians, nurse practitioners) serve as a potential resource in the prevention of cannabis-impaired driving. However, there lacks explicit guidance on the extent of their duties and responsibilities to address this situation. By comparison, a variety of ethical principles have been developed for primary care practice on alcohol-impaired driving. The realities between alcohol versus cannabis consumption and detection differ greatly in practical norms and range of relevant implications such that not all ethical considerations can be transferable [7–11]. Legalization status, accuracy of drug detection, and public policies are just some considerations that differ between the two. These factors can affect the moral and practical landscape for adequately addressing cannabis-impaired driving.

The aim of this critical interpretive review is to provide a broad overview of ethical considerations for primary care providers (including general practitioners) on cannabis-impaired driving [12]. It is presented as general considerations involving the duty to inform, provide care, or report, with specific considerations for potentially vulnerable populations. Ethical conflicts will be considered, such as when is the appropriate time to break patient-clinician confidentiality in the interests of ensuring safety. These recommendations are informed by legal and public policy, professional norms, and codes of conduct for primary care practice, which can differ based on country and jurisdiction.

## Findings: knowledge synthesis of ethical considerations in cannabis-impaired driving

### Considerations for the general population

This section is meant to guide practitioners in the decision-making process, and what the duty to inform, provide care, or report may look like for cannabis-impaired driving in the general population. Further considerations examine when these duties conflict with the practitioner’s code of conduct, such as maintaining confidentiality or respecting the autonomy of patients. Although the recommendations may not be applicable for all patients, this guide serves as a high-level discussion of the ethical principles involved in cannabis-impaired driving.

#### Duty to inform

The duty to inform can be defined as the legal obligation for practitioners to provide information to the patient without explicit request or permission in the interests of ensuring the patient’s well-being [13–15]. Practitioners should convey the exact legal consequences of cannabis-impaired driving, as they are regulated differently in different jurisdictions. For example, many jurisdictions require zero drug levels in saliva for new and commercial drivers [16]. Practitioners should also convey the differing side-effects of cannabis use compared to alcohol or other drug use. The psychoactive cannabis ingredient THC is lipid-soluble rather than water-soluble [17]. Thus, the duration and threshold of cognitive impairment can differ greatly for each person depending on their age, sex, consumption habits, and form of cannabis product used [17]. Some epidemiological studies suggest that lower concentrations of whole blood THC (≤2ng/mL) may not be associated with an increased risk of collision [18]. It is also important to convey that the extent of impairment can vary for the patient each time they consume cannabis and that patients assume this risk when driving.

In addition, the technical complexity of cannabis detection should be discussed. Adequate and accurate on-site biochemical detection of Δ(9)-THC in blood, urine, or saliva remains an issue to accurately quantify the extent of impairment [19]. Traces of cannabis can be detected in the body for days or weeks after intoxication, even after its psychoactive effects have passed, which is a key consideration for frequent drivers [20]. Practitioners have a responsibility to convey the specific harms of cannabis-impairment driving. These harms include delayed reaction time in operating motor vehicles, impaired road visibility, and compromise in judgment of road distance or the driving behavior of others [20]. Finally, the risks of impaired driving should be discussed. Drivers with THC in their body are twice as likely to be involved in a fatal car crash than drivers without alcohol or drugs, with the risk of collision increasing as serum THC concentration increases [20,21].

#### Duty to provide care

The duty to provide care is embodied as acting responsibly and prudently to protect the patient from unnecessary harm, through medical skill, counseling, or otherwise. Care in the context of patients who drive while impaired by cannabis entails a thorough assessment and prevention of any driving risks before impairment occurs, while respecting the autonomy of patients in their cannabis use. As with alcohol use, cannabis can be used infrequently or at low doses such that it does not affect driving skill or lead to addictive behavior. Unlike alcohol use, the signs of cannabis misuse and impairment standards are not as well established or detected [20]. In addition, awareness or discussion of these issues may not be common practice for the patient. Thus, some responsibility falls on the primary care provider to assess the knowledge base of the patient on these matters using probing questions and self-reflective discussion.

When cannabis misuse is suspected, practitioners should ask patients about their driving and cannabis habits, with follow-ups needed for at-risk populations [21]. Lower-risk and infrequent uses of cannabis should be distinguished from problematic uses that can lead to poor decision-making abilities, addictive behaviors, or usage of cannabis in situations where driving may be needed afterwards. Practitioners should also inquire about coincident cannabis use alongside alcohol or other drugs and convey the possible combined psychoactive impacts on motor vehicle operation and cognitive function. To provide adequate care, practitioners should ask about the reasons for cannabis use and treat any underlying conditions associated with its use, with the goal to reduce the patient’s dependency on cannabis to manage these conditions. For example, brief counseling may be provided to patients who rely on cannabis consumption as a coping mechanism for emotional issues, with a referral to specialists when patients are unable to cease use prior to driving [21]. Lastly, practitioners should discuss with patients some tailored harm reduction strategies, such as making a plan of cannabis consumption or securing a designated driver.

Potential barriers to sufficient care are the depth of knowledge conveyed and relationships formed in primary care practice. Given the limited time that practitioners have with patients, it may be unrealistic to sufficiently inform every patient on cannabis-impaired driving or provide adequate care if the needs of patients are beyond the scope of the practitioner. Physicians still have a duty to provide care on this issue, thus, it is recommended to quickly screen patients on cannabis habits to determine the potential dangers of engaging in cannabis-impaired driving.

#### Duty to report

The duty to report is a way for practitioners to fulfill beneficence by stopping potentially harmful situations. This duty is often a professional obligation set by governing bodies of the jurisdiction or medical institution. Generally, reporting cannabis-impaired driving to legal authorities should occur as a last resort or when someone is in imminent danger. Practitioners have an ethical and legal obligation to maintain patient confidentiality, but there are some instances where confidentiality may be compromised in the interests of protecting the patient or public safety [22]. For example, a potentially dangerous situation can occur when the patient acts in voluntary ways that are knowingly unsafe, such as choosing to drive impaired when other safer modes of transportation are accessible [22]. The practitioner is also accountable for recognizing instances where a patient’s actions may not align with self-reported behavior, such as downplaying the effects of cannabis use because the patient does not ‘sense’ an impairment of driving ability. Driving is legally considered a privilege and can be revoked when there is a clear compromise of driving ability. Cannabis-impaired driving should be considered a public health threat, especially when it becomes normalized behavior.

Lastly, there remains the decision whether to report to public health agencies or justice agencies, which will depend on the severity of the situation, whether someone is in imminent danger, and jurisdiction-specific laws. Some jurisdictions obligate the practitioner to report impaired driving to specific authorities, while others prohibit disclosing confidential information without explicit consent [22]. When reporting is authorized by law, practitioners can face civil or criminal liability for unauthorized disclosure to legal authorities when the situation is not dire [22]. On the other hand, when there is no legal obligation to report, practitioners would be free from legally liability if they choose *not* to report, even if the practitioner knows about a potentially dangerous situation [22].

The lack of a uniform legislation might entice primary care providers to report use of cannabis without facing the risk of legal repercussions, since some jurisdictions may allow disclosure of personal information without consent. However, a major consequence for practitioners who report could be a loss of patient trust, which may prompt the patient to withhold disclosure of future health issues and risky behaviors that could endanger the wellbeing of the patient or others. This leaves the question whether jurisdictional differences could eventually act as a deterrent to prevent primary care providers from reporting to authorities due to variance in criminal liability. As cannabis produces different pharmacological effects on different people, it could become difficult for professionals to report misuse with certainty. In lieu of these considerations, the general framework for practitioners is thus to: (1) assess and inform about the risks of cannabis-impaired driving, (2) provide care to minimize harmful consequences of impaired driving, and (3) report instances of significant driving abuse only when necessary to protect third parties from serious harm.

### Considerations for potentially vulnerable populations

**Figure 1.**
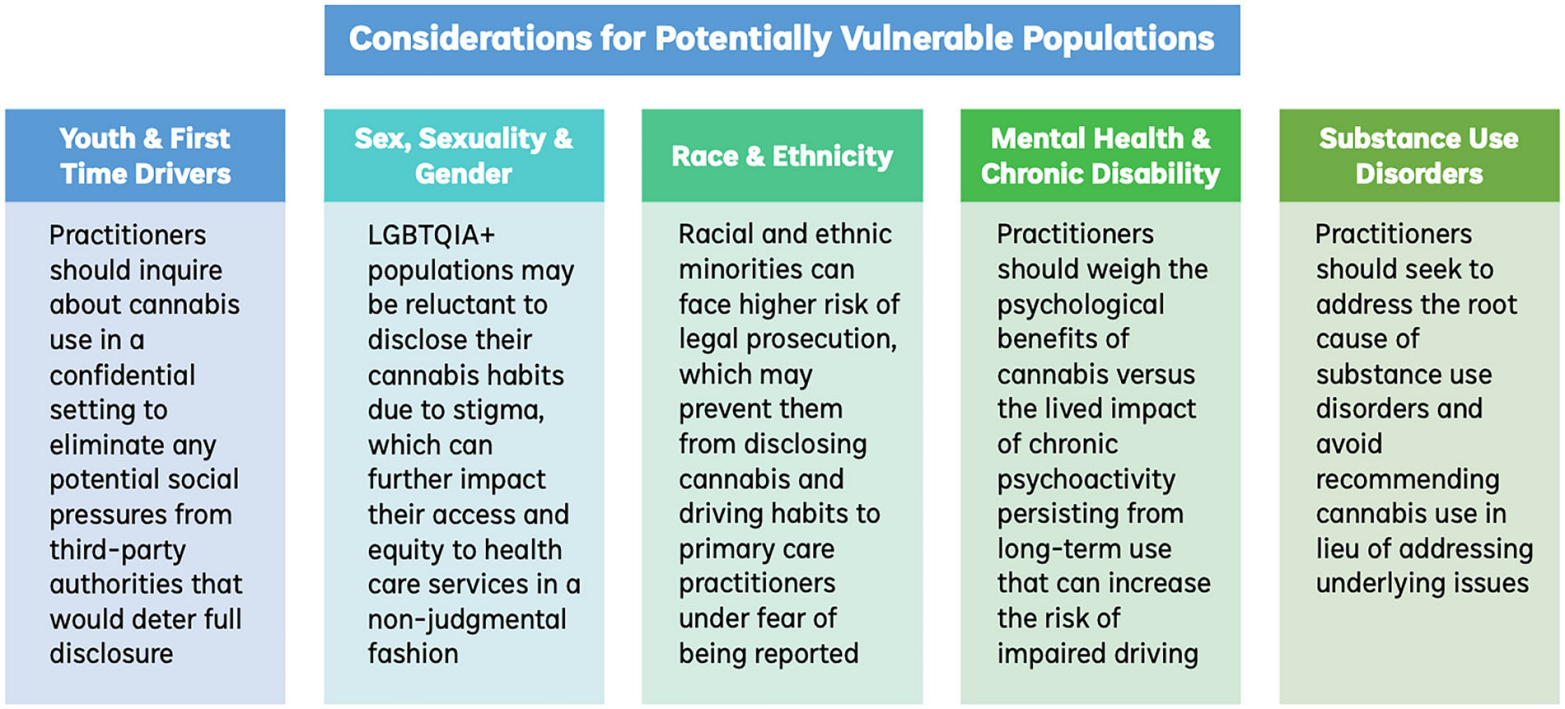
Considerations for potentially vulnerable populations.

When addressing cannabis-impaired driving, practitioners should consider exceptional ethical implications, where a greater risk of harm or standard of care may differ for potentially vulnerable populations. Such populations considered in the ethical analysis include youth and first-time users; sex, sexual and gender minorities; racial and ethnic minorities; people with substance use disorders; and those with mental health disabilities or co-morbidities that rely on prolonged cannabis use to treat their conditions. Here we consider if long-term cannabis use will affect the decision-making process in the practitioner’s duty to inform, report, or provide care, with a summary of the considerations outlined in Figure 1.

#### Youth & first-time user considerations

Drugged driving is defined as driving a vehicle while impaired due to the intoxicating effects of recent drug use, including cannabis [23]. Rates are greatest among adolescents and young adults below 25 years of age, with young drivers at 3 times greater risk than experienced drivers to die while drugged driving [24]. Studies have shown that increased plasticity of the brain during youth can increase the risk for long-term cognitive impairment compared to other age groups [25]. Chronic cannabis use has also been associated with broad neuropsychological decline across various domains of functioning [25]. It could lead to developmental disadvantages in attention and memory post-adolescence and the development of schizophrenia or other mental disorders, with neurocognitive issues persisting even after cannabis use is stopped [25].

First-time users may also lack awareness of their tolerance limits or be more sensitive to the psychoactive effects of cannabis [26]. Exacerbated anxiety, and other psychological impairments are often experienced alongside the psychoactive effects of cannabis use [26]. At the same time, youth or first-time users may be unaware of driving impairment or perceive cannabis-impaired driving to be less risky or harmful than alcohol or drug-impaired driving [25,26]. This may be due to the legalized status of cannabis in many jurisdictions, general acceptance of cannabis use in mainstream society, and technical challenges with on-road detection by law enforcement [25,26]. As youth also have greater non-drugged accident rates than non-youth (given their lack of driving experience and tendency for risk-taking behavior), they are one of the most at-risk demographics for detrimental cannabis-impaired driving.

For these populations, practitioners should inquire about cannabis use and habits in a private and confidential setting to eliminate any potential social pressures from third-party authorities that would deter the youth or first-time user from full disclosure. If the patient drives, practitioners should advise their patient to abstain from driving for at least 6 h after using cannabis and not to ride as a passenger with a cannabis-impaired driver [27,28]. Further abstinence should be advised if patients still feel the psychoactive effects of cannabis or used cannabis alongside other drugs. It is important to emphasize the legal consequences of cannabis-impaired driving, which differs depending on the jurisdiction, but often entails stricter consequences for youth and new drivers such as greater surveillance or loss of license.

Lastly, confidentiality is a key ethical principle to maintain trust in the patient-practitioner relationship. However, the duty to report can sometimes supersede this principle in certain situations. For example, if the youth is being provided cannabis by adults as a means of treating pain or psychological symptoms but cannabis or cannabinoids are not indicated medically [25]. This would fall under the domain of child abuse and neglect, and the role of a practitioner should be treated as such [25]. Another instance when confidentiality may be broken to the parents, but not necessarily reported to law enforcement, is when the youth shows disregard for safe driving protocols or misuse of cannabis while driving, despite the practitioner’s attempts at educating the patient of the risks [25].

#### Sex, sexuality, and gender considerations

The demographic distribution of cannabis consumption is varied in the LGBTQIA + community, with the proportion of cannabis use twice as high among bisexual, homosexual, or non-heterosexual populations than heterosexual counterparts [29]. Although women typically consume cannabis less frequently than men, U.S. and Australian studies found that bisexual women and men use cannabis 2 to 7 times more than their heterosexual counterparts [30]. Cannabis use has also been associated with social support, gay community involvement, coming out to family, and managing depression or other mental health issues [30]. However, LGBTQIA + populations may be more reluctant to disclose their cannabis habits if it points to stigma in belonging to a sexual or gender minority, which can further impact their access and equity to health care services in a non-judgmental fashion [30].

Among pregnant women, the rate of cannabis use has been steadily increasing since legalization, whether medicinal or recreational [25]. Commonly cited reasons for use include alleviating nausea, stress management, and relief from negative mood as a natural alternative to prescription medications that may be contraindicated during pregnancy [25]. Pregnant women may also use cannabis under the belief that it poses little or no risk to the fetus [25]. However, some evidence suggest that chronic *in utero* exposure to cannabis may lead to lower birth weight and preterm delivery at birth, or memory issues, attentional problems, hyperactivity, and impulsivity in early childhood [25]. For these populations, the usage of cannabis may not be medically-justified, and practitioners should strongly counsel against cannabis use in the context of driving [31]. Regardless, it is important to address the patient’s reasons for cannabis use and offer alternatives to safe management of these concerns. In doing so, practitioners should address beliefs countered by evidence, by informing and helping interpret medical evidence for the patient.

#### Race and ethnicity considerations

The ethical principle of justice, embodied as differential behavior to ensure equity and fairness, should be considered for racial and ethnic differences, as the policing of cannabis-impaired driving is tied to North American inequalities in how cannabis consumption is regulated between racial and ethnic minorities [32]. Black drivers typically have a higher likelihood of being stopped and thus testing positive for cannabinoids by law enforcement [32]. Despite legalization, racial minorities like Black, Hispanic, Indigenous and some Asian subpopulations are more likely than White populations to experience negative legal consequences of cannabis use [33]. These populations can face legal prosecution, which may prevent them from disclosing cannabis and driving habits to their practitioners under fear of being reported [33]. In communities where frequent cannabis use is normalized, users may not perceive cannabis-impaired driving to be as dangerous [34]. They may also be less inclined to seek out help from professionals except under extreme circumstances [34].

Normalized cannabis use in Indigenous communities has been attributed to the realities of intergenerational trauma, unresolved grief, lack of social and health support, comparisons to ‘harder’ drug use, and perceived racism [34]. Driving is also a necessary mode of transportation in reserves and remote locations without public transportation infrastructure, thus potentiality increasing the rate of cannabis-impaired driving [34]. Indigenous communities can also face a shortage of primary care practitioners who are trained in addressing the unique situations of the community [35]. In taking a nuanced approach by using analogies to violence-informed care is key to mitigating risks of cannabis-impaired driving while emphasizing confidentiality, even if the patient may be reluctant to disclose cannabis use. Practitioners can also prioritize referrals to harm reduction resources (e.g. public health programs on substance addiction) over reporting cannabis misuse to legal authorities, unless the patient poses immediate danger, or the practitioner is legally required to do so.

#### Mental health & chronic disability considerations

Since cannabis legalization, the production and advertisement of over-the-counter cannabis products have increased, often targeting those with mental health concerns as a way to self-manage symptoms [25]. Cannabis use has also increased during the COVID-19 pandemic [36,37]. Even without an explicit diagnosis, commonly cited reasons for increased cannabis use include anxiety, boredom, stress, and to relax [29]. However, the use of cannabis-related products for the treatment of mental health disorders (e.g. Tourette’s, depression, attention deficit hyperactivity disorder, post-traumatic stress disorder, psychosis, anxiety) is not well supported by scientific research [38]. Cannabis use can also lead to induced psychosis, creating mental health issues in individuals who are otherwise healthy, or lead to schizophrenia or bipolar disorder [39]. On the other hand, nabilone or pharmaceutical grade tetrahydrocannabinol (THC) has been shown to provide a small benefit for anxiety symptoms among patients with chronic non-cancer pain and multiple sclerosis, while prolonged cannabis use has been shown to have medicinal benefits in the management of comorbid conditions like chronic pain, nausea, and muscle spasms [25,38].

For regular drivers who use cannabis to alleviate symptoms of mental illness, it is recommended to reduce or prevent dependency on cannabis as the primary method to manage mental health symptoms. This may mean prescribing other non-psychoactive medication or referring patients to psychotherapy. Otherwise, practitioners should weigh the psychological benefits of cannabis versus the lived impact of chronic psychoactivity persisting from long-term use that can increase the risk of impaired driving. It is important to remember that the dosage and duration of psychoactive cannabis effects can differ depending on a patient’s drug use history, tolerance level, and underlying mental health. Thus, practitioners should help patients to establish a personalized cannabis usage plan for ensuring driving safety and optimizing health. Recall that practitioners have a duty to report suspected cases of cannabis misuse that can lead to serious driving harm or injury.

#### Substance use disorder considerations

Some practitioners prescribe medical cannabis to patients with substance use disorders, as it has proven useful as a safer alternative to illicit substances [25]. On the other hand, other studies have shown that using cannabis can exacerbate addictive behaviors, making its use contraindicated for individuals wishing to curb behavioral issues in substance use disorders [25]. Prescribed cannabis can also be misused in practice, and practitioners should consider that those with existing substance use disorders may have a higher likelihood of misuse. To distinguish between these instances, practitioners should seek to address the root cause of substance use disorders and to avoid recommending cannabis use in lieu of addressing underlying issues.

Recall that in the context of driving, the use of cannabis alongside other drugs can lead to severe sensory, motor, and cognitive impairments. Even if the patient does not exhibit adverse reactions or disordered substance use to cannabis alone, the risk of combined drug use, including alcohol, increases significantly. Therefore, driving should be discouraged altogether in the interests of protecting patient and public safety if the patient chooses to use cannabis regularly to manage substance use disorders. Patients may be comfortable discussing legal cannabis use but may be reluctant to disclose coincidental/illicit drug use alongside driving habits. Thus, practitioners should take extra caution to assess the dosage, frequency, and degree of psychoactivity, emphasizing the need to quantify the extent of general impairment rather than policing cannabis or illicit drug use.

## Conclusion

The ethical approach for primary care practitioners in addressing cannabis-impaired driving can be summarized as the duty to always inform, provide care through prevention and harm reduction strategies, and report when necessary. The prevention of cannabis-impaired driving should not fall on the sole responsibility of primary care practitioners. This review offered a high-level discussion of the ethical considerations in cannabis-impaired driving, noting that specific recommendations will depend upon the legal and policy designations of individual jurisdictions. For example, the availability of specific screening and interventions will depend on the available resources and time commitments of the primary care provider, amongst other factors.

Nonetheless, the following recommendations are made to improve public safety regarding cannabis-impaired driving: (1) an increase in public health programs that focus on conveying the risks and harms in cannabis-impaired driving, tailored to youth and other potentially vulnerable populations; (2) a prioritization of preventative rather than punitive health policy measures to decrease the incidence of cannabis-impaired driving, and (3) improved tracking of cannabis-impaired driving trends, based on demographics such as race, gender, geographical location, socioeconomic status, and cannabis legalization status. Ultimately, the practitioner should manage cannabis-impaired driving in a way that fosters the therapeutic relationship in patient-centered care, through motivational discussions, collaboration with specialists, skills for self-management, patient empowerment, and support.

## Data Availability

Data sharing is not applicable to this article as no new data were created or analyzed in this study.

## Appendix A: Methods

As such guidance extended beyond the scope of a systematic review, an ethical analysis in the form of a critical interpretive review was undertaken instead, using a systematic approach to determine the appropriate action to a given situation with evidence to substantiate its claims [10]. We used the review process described by McDougall [12]. The search strategy was designed to answer the research question: What are some ethical concerns for primary care providers to consider when cannabis-impaired driving is suspected? Google Scholar, PubMed, Web of Science, and Scopus databases were searched in December 2021 using MeSH terms (when available) and keywords related to cannabis, impaired driving, ethics, and primary care. The most recent publications and literature relevant to a legalized cannabis context were prioritized and snowballed according to relevance. The search was then repeated for different potentially vulnerable populations of interest. Topic substitutions in the search strategy were used as needed when the scope of ethical analysis was insufficient (i.e. analyzing primary care ethics of alcohol-impaired driving), while synthesizing ethical considerations relevant to the context of cannabis-impaired driving. A summary of the search process is outlined in Appendix A. The resulting evidence was synthesized as recommendations for primary care practice.

Search & Screening Methods:

Each search was conducted on the selected databases using the search strategy provided. Scopus returned 23 results, PubMed (without MeSH terms) returned 20 results, PubMed with MeSH terms returned 218 results, Google Scholar search #1 returned 632 results, Google Scholar search #2 returned approximately 157,000 results, Embase (without EMTREE terms) returned 11 results, and Embase with EMTREE terms returned 51 results. All results were screened, except for Google Scholar, where the first 300 results were screened. For all results, the title and abstract were screened to see if the topic of study matched all the eligibility criteria of: (1) the topic was pertaining to cannabis consumption, drugged impairment while driving, or cannabis-impaired driving; (2) the manuscript explored an ethical or public health analysis/interpretation of cannabis consumption, such as risks, harms, duties, public health policy implications; and (3) the article included moral considerations from the perspective of primary care physicians or practitioners. The search was repeated with keywords indicative of specific subpopulations of focus, i.e. “race” or “ethnicity” or “indigenous” as keywords for topics on cannabis-impaired driving in Indigenous populations. A snowball procedure was used for articles that had highly relevant topics, where the citation list was screened to identify other relevant articles. All article types were considered, including reviews and commentaries.

## Appendix B: Search results & screening methods

**Date Search Performed**: December 6, 2021

**Table:**

Databases	Keywords
Scopus	( TITLE-ABS-KEY ( cannabis-impaired AND driving ) AND TITLE-ABS-KEY ( confidentiality OR physician OR responsibilities OR duty OR to OR report OR harms OR risks ) )
Pubmed	(cannabis-impaired AND driving) AND (confidentiality OR physician OR responsibilities OR duty OR to OR report OR harms OR risks)
Pubmed with MeSH terms	("cannabis"[MeSH Terms] OR "cannabis"[All Fields] OR "cannabi"[All Fields] OR "cannabis s"[All Fields]) AND ("impair"[All Fields] OR "impaired"[All Fields] OR "impairement"[All Fields] OR "impairements"[All Fields] OR "impairing"[All Fields] OR "impairment"[All Fields] OR "impairments"[All Fields] OR "impairs"[All Fields]) AND ("automobile driving"[MeSH Terms] OR ("automobile"[All Fields] AND "driving"[All Fields]) OR "automobile driving"[All Fields] OR "driving"[All Fields] OR "drive"[MeSH Terms] OR "drive"[All Fields] OR "drives"[All Fields] OR "drivings"[All Fields]) AND ("confidential"[All Fields] OR "confidentiality"[MeSH Terms] OR "confidentiality"[All Fields] OR "confidentially"[All Fields] OR ("physician s"[All Fields] OR "physicians"[MeSH Terms] OR "physicians"[All Fields] OR "physician"[All Fields] OR "physicians s"[All Fields]) OR ("responsabilities"[All Fields] OR "responsability"[All Fields] OR "responsibilities"[All Fields] OR "responsibility"[All Fields] OR "responsible"[All Fields] OR "responsibles"[All Fields]) OR "duty"[All Fields] OR ("toxicity"[MeSH Subheading] OR "toxicity"[All Fields] OR "to"[All Fields]) OR ("reportable"[All Fields] OR "reporting"[All Fields] OR "reportings"[All Fields] OR "research report"[MeSH Terms] OR ("research"[All Fields] AND "report"[All Fields]) OR "research report"[All Fields] OR "report"[All Fields] OR "reported"[All Fields] OR "reports"[All Fields]) OR ("harmed"[All Fields] OR "harmful"[All Fields] OR "harmfulness"[All Fields] OR "harming"[All Fields] OR "harms"[All Fields]) OR ("risk"[MeSH Terms] OR "risk"[All Fields])) **Translations** **cannabis:** "cannabis"[MeSH Terms] OR "cannabis"[All Fields] OR "cannabi"[All Fields] OR "cannabis’s"[All Fields] **impaired:** "impair"[All Fields] OR "impaired"[All Fields] OR "impairement"[All Fields] OR "impairements"[All Fields] OR "impairing"[All Fields] OR "impairment"[All Fields] OR "impairments"[All Fields] OR "impairs"[All Fields] **drive:** "automobile driving"[MeSH Terms] OR ("automobile"[All Fields] AND "driving"[All Fields]) OR "automobile driving"[All Fields] OR "driving"[All Fields] OR "drive"[MeSH Terms] OR "drive"[All Fields] OR "drives"[All Fields] OR "drivings"[All Fields] **confidentiality:** "confidential"[All Fields] OR "confidentiality"[MeSH Terms] OR "confidentiality"[All Fields] OR "confidentially"[All Fields] **physician:** "physician’s"[All Fields] OR "physicians"[MeSH Terms] OR "physicians"[All Fields] OR "physician"[All Fields] OR "physicians’s"[All Fields] **responsibilities:** "responsabilities"[All Fields] OR "responsability"[All Fields] OR "responsibilities"[All Fields] OR "responsibility"[All Fields] OR "responsible"[All Fields] OR "responsibles"[All Fields] **to:** "toxicity"[Subheading] OR "toxicity"[All Fields] OR "to"[All Fields] **report:** "reportable"[All Fields] OR "reporting"[All Fields] OR "reportings"[All Fields] OR "research report"[MeSH Terms] OR ("research"[All Fields] AND "report"[All Fields]) OR "research report"[All Fields] OR "report"[All Fields] OR "reported"[All Fields] OR "reports"[All Fields] **harms:** "harmed"[All Fields] OR "harmful"[All Fields] OR "harmfulness"[All Fields] OR "harming"[All Fields] OR "harms"[All Fields] **risk:** "risk"[MeSH Terms] OR "risk"[All Fields]
Google Scholar	(cannabis-impaired AND driving) AND (confidentiality OR physician OR responsibilities OR duty OR to OR report OR harms OR risks)
	((cannabis OR alcohol) impaired AND driving) AND (confidentiality OR physician OR responsibilities OR duty OR to OR report OR harms OR risks)
Embase/Ovid	(cannabis-impaired and driving and (confidentiality or physician or responsibilities or duty or to or report or harms or risks)).mp. [mp = title, abstract, heading word, drug trade name, original title, device manufacturer, drug manufacturer, device trade name, keyword heading word, floating subheading word, candidate term word]
Embase/Ovid with EMTREE terms	(exp/cannabis and exp/drive) and (exp/ethics or exp/risk)

## Appendix C: Summary of key findings in select eligible studies

**Table:**

Year	Author	Title & Key Findings
2007	Bédard, M., Dubois, S. & Weaver, B.	The Impact of Cannabis on Driving Of the 32,543 drivers tested, 1,647 (5%) tested positive for cannabis. Drivers who tested positive were generally younger, male, and had a poorer driving record in the past three years. A greater proportion of drivers who tested positive for cannabis had a DRF related to speeding or erratic/reckless driving. The crude OR between DRFs and cannabis was 1.39 (99% CI = 1.21-1.59). After adjusting this association for age, sex, and driving record the OR for THC was 1.29 (99% CI = 1.11-1.50). Younger age and poorer driving records, but not sex, were also associated with a higher risk of a reported DRF
2018	Lee, J., Abdel-Aty, A. & Park, J.	Investigation of associations between marijuana law changes and marijuana-involved fatal traffic crashes: A state-level analysis Full legalization and decriminalization of marijuana have significant effects on marijuana-related crashes in all states.
2013	Li, G., Brady, J. E. & Chen, Q.	Drug use and fatal motor vehicle crashes: A case-control study Overall, 31.9% of the 737 cases and 13.6% of the 7719 controls tested positive for at least one non-alcohol drug, yielding a crude odds ratio of 2.98 (95% CI: 2.51, 3.53). As expected, male drivers and drivers aged 21 to 34 years or 65 years and older were at significantly increased risk of fatal crash involvement. The heightened risk of fatal crash involvement associated with drug use existed in each age group and in both sexes. Although drivers aged 35–44 years and female drivers appeared to be at particularly excess risk, the estimated odds ratios of fatal crash involvement did not vary significantly with age and sex. Polydrug use, defined as use of two or more non-alcohol drugs, was associated with a 3.4-fold increased risk of fatal crash involvement. The estimated odds ratios of fatal crash involvement associated marijuana was 1.83 [95% confidence interval (CI): 1.39, 2.39].
2016	Rogeberg, O. & Elvik, R.	The effects of cannabis intoxication on motor vehicle collision revisited and revised: Cannabis and motor vehicle collision risk The revised estimate from the studies used in Asbridge et al. was in line with the results from the expanded meta-analysis in study 2, lying between the pooled odds from the mixed-effects model of 1.36 (CI = 1.15–1.61) and the pooled odds from the PEESE meta-regression of 1.22 (meta-regression model, CI = 1.1–1.36). Acute cannabis intoxication is associated with a statistically significant increase in motor vehicle crash risk. The increase is of low to medium magnitude.
2020	Brubacher, J. R., Chan, H. & Staples, J. A.	Cannabis-impaired driving and Canadian youth Among Canadian National Cannabis Survey respondents aged 15 to 24 years, about one in four reported using cannabis in the previous 3 months, 14% reported driving within 2 h of using cannabis, and 12% reported being a passenger in a vehicle driven by someone who had used cannabis in the previous two hours. Among Canadian youth who use cannabis frequently, 80% of males and 75% of females reported that in the previous 30 days they had been in a car driven by someone (including themselves) who had recently used marijuana or other drugs and 64% of males and 33% of females reported being ‘intoxicated’ with marijuana while operating a vehicle. Cannabis impairs the psychomotor skills required for safe driving. Cannabis contains over 60 cannabinoids, but most impairing effects are caused by the main psychoactive compound, Δ-9-tetrahydrocannabinol (THC) (18). After taking cannabis, most people experience the desired effects of euphoria and relaxation, although some develop anxiety, panic, or even paranoia. After taking cannabis, drivers in traffic ‘weave’ more, sometimes to the point of being unable to stay within the traffic lanes (28). They also have difficulty maintaining a constant speed or a uniform gap between themselves and a lead vehicle (28). In simulator studies, drivers tend to slow down after using cannabis, have delayed reactions when responding to a sudden hazard, and are more likely to crash (29–32). This evidence suggests that people who drive after using cannabis might have a large increase in collision risk. Epidemiological studies show a modest increase in crash risk after cannabis use. Several recent meta-analyses all concluded that cannabis use increases crash risk, with odds ratios (ORs) ranging from 1.36 to 2.66 (34,35). Driving simulator studies and epidemiologic analyses suggest that cannabis use increases motor vehicle crash risk. Young, inexperienced drivers may be at a particularly high risk of crashing after using cannabis.
2017	Fischer, B. et al.	Lower-Risk Cannabis Use Guidelines: A Comprehensive Update of Evidence and Recommendations Cannabis use acutely impairs key executive functions critical for driving, including cognition, attention, memory, decision-making, and psychomotor functioning. This occurs in a dose-dependent way, although the magnitude and persistence of impairments may vary with use patterns, THC concentration, tolerance, metabolism, and other factors. Some of these impairments have been found to persist after acute intoxication, particularly in chronic users. Following cannabis intake, peak THC plasma concentrations (around 100 ng/mL) are usually reached within approximately 5 to 30 min and generally taper off approximately 2 to 4 h later. However, intoxication and cognitive impairments may persist beyond THC plasma concentration peaks, yet typically clear within approximately 3 to 6 h. Higher THC or other cannabinoid concentration or ingested cannabis products (with an extended absorption period) can have more pronounced and persistent effects. Although these effects are based on the typical pharmacokinetics of THC, they may vary with inhalation intensity, lung capacity, and other factors.
